# Cicha Strefa płuc − Zastosowanie Testu Wypłukiwania Azotu Metodą Wielokrotnych Oddechów (MBNW) W Diagnostyce Chorób Drobnych Dróg Oddechowych u Dzieci − Doniesienie Wstępne Na Podstawie Piśmiennictwa i Doświadczenia własnego

**DOI:** 10.34763/devperiodmed.20172104.369379

**Published:** 2018-01-02

**Authors:** Katarzyna Walicka-Serzysko, Magdalena Postek, Dorota Sands

**Affiliations:** 1 Klinika i Zakład Mukowiscydozy Instytutu Matki i Dziecka w Warszawie, Warsaw Polska; 2 Centrum Leczenia Mukowiscydozy w Dziekanowie Leśnym, Dziekanów Leśny Polska

**Keywords:** dzieci, gaz obojętny, funkcja płuc, drobne drogi oddechowe, dystrybucja wentylacji, badania czynnościowe płuc, children, inert gas, lung function, small airways, ventilation distribution, pulmonary function tests

## Abstract

Drobne drogi oddechowe są miejscem powstawania zmian patologicznych w przebiegu wielu chorób jak np. astma lub mukowiscydoza, często już we wczesnym ich stadium. Ta część dróg oddechowych jest jednak pomijana w konwencjonalnych badaniach czynnościowych układu oddechowego i z tego powodu często nazywana jest „cichą strefą płuc”. W niniejszej pracy przedstawiono podstawy teoretyczne testu wypłukiwania azotu metodą wielokrotnych oddechów (MBNW − ang. multi-breath nitrogen washout) w diagnostyce chorób drobnych dróg oddechowych. Omówiono zagadnienia techniczne związane z przygotowaniem pacjentów pediatrycznych do przeprowadzenia badania oraz przebieg wykonywania testu. Możliwości zastosowania klinicznego opisanej metody stanowią nadal przedmiot wielu badań oraz budzą nadzieje na wypełnienie luki w testach czynnościowych drobnych dróg oddechowych. Ze względu na zaangażowanie autorów w diagnostykę i leczenie chorych na mukowiscydozę w pracy opisano również doświadczenia własne dotyczące wykorzystania tego badania w tej grupie pacjentów. Obecnie metoda znajduje się w fazie intensywnie prowadzonych analiz związanych z wykryciem wczesnych stadiów choroby oskrzelowo-płucnej w przebiegu mukowiscydozy, kiedy jeszcze wyniki innych badań czynnościowych są prawidłowe lub niemożliwe do wykonanie z uwagi na wiek pacjenta. Korelacja z metodami obrazowymi (tomografia komputerowa klatki piersiowej) i nasileniem zmian strukturalnych może w przyszłości ograniczyć liczbę wykonywanych badań radiologicznych, a tym samym zmniejszyć narażanie pacjenta na promieniowanie jonizujące. Wprowadzenie testów oceniających funkcję płuc u niemowląt i dzieci przedszkolnych z mukowiscydozą i innymi chorobami drobnych dróg oddechowych może zmodyfikować postępowanie kliniczne i poprawić rokowanie.

## Abstract (en)

Small airways are the site of pathological changes often in an early stage in many diseases such as asthma or cystic fibrosis. However this part of the airways is overlooked in conventional respiratory function tests and it is therefore often referred to as “silent lung zone”. This paper presents the theoretical background of MBNW multi-breath nitrogen washout in the diagnosis of minor respiratory diseases. The technical issues related to the preparation of pediatric patients for the test. Clinical applications of the MBNW test results are still subject to a number of studies. There is hope for filling the gaps in the small airway function tests. Due to the authors’ involvement in the diagnosis and treatment of patients with cystic fibrosis, their own experience in the use of this study was also described. Currently, the method is in the phase of intensive analysis for the early diagnosis of lung disease in cystic fibrosis, when still other functional tests are in normal range or impossible to perform due to patient age. Correlation with medical imaging methods (chest computed tomography) and the severity of structural changes may in future limit the amount of radiology tests. In addition this can reduce the patient’s exposure to ionizing radiation. Introduction of lung function tests such a MBW in infants and preschool children with cystic fibrosis and other minor respiratory diseases may modify clinical management and improve prognosis.

## Wstęp

„Cichą strefą płuc” (ang.” silent zone”) nazywane są obwodowe drogi oddechowe poniżej siódmej generacji, nie posiadające ściany chrzęstnej, których wewnętrzna średnica nie przekracza 2 mm. Swoją nazwę zawdzięczają brakiem możliwości ich pełnej oceny na podstawie dotychczas wykonywanych badań czynnościowych układu oddechowego, w tym przede wszystkim spirometrii. Wiadomo jednak, że w niektórych jednostkach chorobowych, takich jak mukowiscydoza (CF − ang. cystic fibrosis), astma, przewlekła obturacyjna choroba płuc (POCHP) wcześnie dochodzi do zaburzeń w drobnych drogach oddechowych. Badania histopatologiczne tkanki płucnej pobranej pośmiertnie oraz podczas biopsji przezoskrzelowych in vivo, potwierdzają udział w patogenezie astmy zarówno centralnych jak i obwodowych dróg oddechowych. Podobnie badania autopsyjne tkanki płucnej chorych na mukowiscydozę opisują zmiany patologiczne w drobnych oskrzelach. Już we wczesnym stadium choroby oskrzelowo-płucnej w badaniach obrazowych uwidaczniane są: pogrubienie ścian oskrzeli, „objaw pułapki powietrznej”, korki śluzowe i rozstrzenie oskrzeli. Ze względu na niewielki wpływ oporu obwodowych dróg oddechowych na ich opór całkowity, wyniki badań spirometrycznych często mieszczą się jeszcze w granicach normy lub ze względu na wiek i brak współpracy z dzieckiem, testy te nie mogą być wykonane. Do niedawna przebieg choroby dróg oddechowych np. w mukowiscydozie lub astmie był monitorowany jedynie na podstawie wyników spirometrii − metody odzwierciedlającej nieprawidłową funkcję dróg oddechowych w zaawansowanym stadium choroby [1].

Wykrycie zmian w obwodowych drogach oddechowych umożliwia wdrożenie odpowiedniego leczenia. Z tego powodu istnieje duża potrzeba stosowania w diagnostyce chorób płuc metod nieinwazyjnych pozwalających na jak najwcześniejsze wykrywanie zaburzeń w zakresie drobnych dróg oddechowych przy jednoczesnym nieskomplikowanym sposobie ich wykonania, umożliwiającym badanie dzieci oraz słabo współpracujących pacjentów dorosłych (np. osób starszych).

Wprowadzenie testu wypłukiwania gazu metodą wielokrotnych oddechów (MBW − ang. multi-breath washout) pozwala na wykrycie nieprawidłowej funkcji dróg oddechowych charakteryzującej się zaburzoną dystrybucją wentylacji oraz patologiczną pułapką powietrzną m.in. u dzieci z astmą lub mukowiscydozą już we wczesnym stadium choroby [1, 2, 3, 4].

## Drobne drogi oddechowe - „Cicha strefa płuc”

Drogi oddechowe pod względem czynnościowym można podzielić na strefę przewodzącą ( nie oddechową), czyli oskrzela i oskrzeliki doprowadzające powietrze do pęcherzyków płucnych oraz strefę oddechową, w której odbywa się wymiana gazowa.

W wyniku dychotomicznych ale niesymetrycznych podziałów tworzą one ok. 23 generacje (pokolenia) [5]. Jednostką wymiany gazowej jest gronko obejmujące trzy rzędy oskrzelików oddechowych, 4-5 rozgałęzień przewodów pęcherzykowych i na końcu każdego z nich woreczek z 10-16 pęcherzykami płucnymi. U dorosłego człowieka ponad 20 000 gronek tworzy ok. 100 m^2^ powierzchni wymiany gazowej pomiędzy wdychanym powietrzem a krwią naczyń włosowatych. Złożona struktura płuc zapewnia efektywne mieszanie gazów i prawidłową dystrybucję wentylacji. Obwodowe drogi oddechowe tworzą oskrzela poniżej siódmej generacji, o wewnętrznej średnicy poniżej 2 mm (u osób dorosłych), nie posiadające ściany chrzęstnej (ryc.1). Stanowią one 95% całkowitej pojemności płuc ale tylko 10-20% całkowitego oporu dróg oddechowych. Konwencjonalne badania czynnościowe płuc dostarczają informacji o dużych oskrzelach pomijając drobne drogi oddechowe.

**Ryc. 1:**
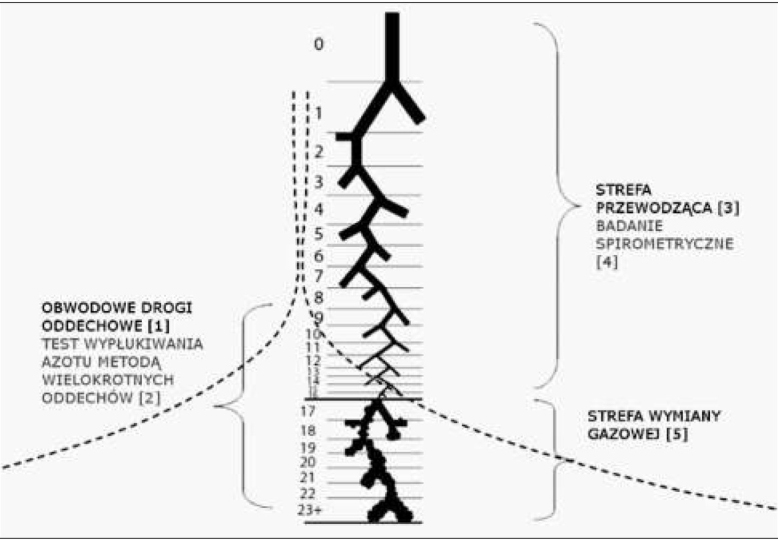
Budowa układu oddechowego: strefa przewodząca (pierwszych 16 generacji): tchawica, oskrzela główne, płatowe, segmentowe, wewnątrzsegmentowe, oskrzeliki końcowe oraz strefa wymiany gazowej (od 17 do 23-24 generacji). Obwodowe drogi oddechowe (tzw. drobne drogi oddechowe − ang. small airways) – oskrzela poniżej siódmej generacji, nie posiadające ściany chrzęstnej i których wewnętrzna średnica nie przekracza 2 mm (u osoby dorosłej) wg [6]. Fig. 1. Respiratory structure: Conductive zone (first 16 generations): trachea, main bronchi, patches, segmental, intrasegmental, bronchial terminal and gas exchange zone (17 to 23-24 generations). Respiratory tract (small airways) − Bronchial below the seventh generation without a cartilage wall and whose inner diameter does not exceed 2 mm (in an adult) [6]. [1] Peripheral respiratory tract, [2] Multiple breath nitrogen washout test, [3] Conductive zone, [4] Spirometry, [5] Gas exhange zone

W strefie przewodzącej dominuje transport gazu za pomocą konwekcji, a liniowa szybkość przepływu gazu jest względnie wysoka. W przypadku obwodowych dróg oddechowych transport oraz wymiana gazów za pomocą konwekcji i dyfuzji molekularnej mają równoważny udział tworząc tzw. „front dyfuzyjno-konwekcyjny”.

Prędkość liniowa gazu w obwodowych drogach oddechowych jest stosunkowo mała, a udział oporu dróg oddechowych i związane z tym faktem ograniczenie przepływu pozostają niewielkie. Z tego powodu zmiany patologiczne w obwodowych drogach oddechowych nie zawsze są wykrywane podczas spirometrii [6].

## Testy wypłukiwania gazu obojętnego − IGW (ang. Inert gas washout)

Pierwsze opisy technik wypłukiwania gazów znacznikowych pojawiły się ponad 60 lat temu.

IGW-wypłukiwanie gazu obojętnego jako metoda pionierska opisana w 1940 roku, umożliwia pomiar heterogenności wentylacji (VI − ang. ventilation inhomogenity) i wnikliwą ocenę funkcji drobnych dróg oddechowych [7]. Jednak pozostała ona niedoceniona i niewykorzystana aż do czasu wprowadzenia zaawansowanych technik analitycznych oraz nowoczesnych metod przetwarzania danych. Obecnie jest uważana za wnikliwą i przydatną metodę oceniającą funkcję drobnych dróg oddechowych. IGW może być mierzone przy użyciu testu wypłukiwania azotu lub innego gazu znacznikowego (np. He, SF_6_) metodą pojedynczego oddechu (SBW − ang. single-breath washout) lub wielokrotnych oddechów (MBW). Wspomniane metody SBW oraz MBW zostały opracowane i po raz pierwszy opisane przez Becklake w 1952 roku. Dzięki rozwojowi techniki jaki nastąpił w XX i XXI możliwe jest wykonywanie analiz w czasie rzeczywistym z jednoczesnym wykorzystaniem zaawansowanych metod obliczeniowych, co umożliwia ocenę nie tylko stopnia homogenności wymiany gazowej, ale również zlokalizowanie toczących się procesów chorobowych. Test wypłukiwania azotu metodą wielkokrotnych oddechów jako wymagający minimalnej współpracy z pacjentem znalazł swoje zastosowanie zwłaszcza wśród pacjentów pediatrycznych. Po wielu latach testów w laboratoriach badawczych metoda ta została zaakceptowana przez grupy ekspertów ERS/ATS i ECFS-CTN [8, 9, 10].

**Ryc. 2:**
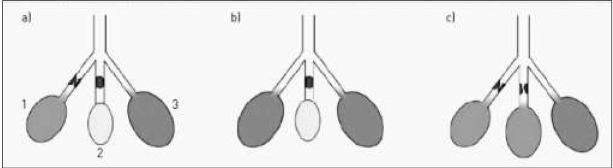
Możliwe efekty leczenia zaostrzenia zmian oskrzelowo-płucnych w przebiegu mukowiscydozy wpływające na niejednorodność wentylacji płuc (LCI − ang. lung clearence index − wskaźnik oczyszczania płuc ) oraz czynnościową pojemność zalegającą (FRC − ang. functional residual capacity) wg [12]: Stan przed leczeniem: 1. Jednostka częściowo zatkana i słabo wentylowana 2. Jednostka całkowicie zatkana i niewentylowana 3. Jednostka w której wentylacja zachodzi bez przeszkód. Przykładowe możliwe wyniki leczenia: 1. W jednostce częściowo zatkanej nastąpiło odetkanie i powrót do prawidłowej wentylacji. 2. Fragment całkowicie zatkany pozostaje bez zmian. W tym wypadku powinno nastąpić zmniejszenie heterogenności wentylacji ( a tym samym poprawa wskaźnika LCI) oraz wzrost FRC. 1. Jednostka częściowo zatkana pozostaje bez zmian. 2. Jednostka całkowicie zatkana została częściowo otwarta. Spowoduje to wzrost FRC i niejednorodności wentylacji ( wzrost LCI). Fig. 2. Possible effects of the treatment of bronchopulmonary changes affecting the heterogeneity of lung ventilation (LCI − lung clearence index and FRC − functional residual capacity [12]: a) Pre-treatment status: 1) partially obstructed and poorly ventilated unit; 2) completely obstructed and unventilated unit; 3) unobstructed unit. b) Possible outcome of treatment of the same three lung units: 1. the partially obstructed unit has been cleared and is now normally ventilated. This should reduce ventilation inhomogeneity (and hence LCI) and increase FRC. c) Another possible outcome of treatment: the unventilated unit has now been partially opened up and is poorly ventilated. This will therefore increase FRC, but will also increase ventilation inhomogeneity, leading to an increase in LCI.

Testy SBW i MBW odzwierciedlają przede wszystkim funkcję drobnych dróg oddechowych - główne miejsce mieszania się powietrza wdechowego z wydechowym. Na niejednorodność wentylacji płuc może wpływać wiele czynników. Najważniejsze z nich to:

− różnice w wymianie gazowej pomiędzy różnymi obszarami płuc,
− sekwencyjne napełnianie i opróżnianie jednostek płuc,
− asymetria w budowie dystalnej części płuc.

Ze względu na wpływ grawitacji i niesymetryczne podziały dróg oddechowych nawet u osób zdrowych występuje niewielkiego stopnia niejednorodność wentylacji płuc. Zaburzenia wymiany gazowej związane z chorobą dodatkowo ją nasilają [6].

W przebiegu mukowiscydozy, a także innych chorób drobnych dróg oddechowych dochodzi do nieodwracalnych zmian w wyniku procesów włóknienia i postępującej destrukcji tkanki płucnej, a także zaburzeń potencjalnie odwracalnych związanych z miejscowym stanem zapalnym i zaleganiem śluzu [1]. Antybiotykoterapia oraz fizjoterapia zmniejszają nasilenie procesów zapalnych w układzie oddechowym, zaleganie śluzu i tworzenie korków śluzowych, ale nie mają wpływu na zmiany utrwalone, takie jak rozstrzenie oskrzeli czy zmiany włókniste [11, 12] (ryc. 2).

## Test wypłukiwania azotu metodą wielokrotnych oddechów (MBWN)

W chorobach układu oddechowego zmiany zachodzące w obwodowych drogach oddechowych powodują niejednorodność wentylacji. Test MBNW z powodzeniem stosowany jest w celu oceny homogeniczności wentylacji stanowi cenne źródło informacji o stopniu oraz lokalizacji niejednorodności wentylacji płuc, a także jest dobrym uzupełnieniem spirometrii, gdyż analizie podlegają w głównej mierze procesy zachodzące na poziomie 8-23generacji oskrzelików o średnicy poniżej 2mm. Wytyczne dotyczące procedur oraz protokół badania zostały przedstawione w najnowszym dokumencie konsensusu ERS/ATS [9, 10].

## Przebieg testu MBWN

W Pracowni Badań Czynnościowych Płuc Centrum Leczenia Mukowiscydozy w Dziekanowie Leśnym test MBNW wykonywany jest przy użyciu urządzenia EXHALYZER® D ECO MEDICS AG. W tym przypadku wykorzystuje się Njako gaz obojętny ulegający wypłu_2_ kaniu (ryc. 3). W celu określenia aktualnego stężenia N_2_ stosuje się technikę pośrednią polegającą na analizie w czasie rzeczywistym stężeń dwutlenku węgla (CO_2_) oraz tlenu O_2_. Kolejnym krokiem jest wyliczanie stężenia N_2_ wg wzoru:

$$1={FO}_{2}+{FCO}_{2}+{FN}_{2}+{FAr}^{⋆}$$

*F oznacza ułamkowe stężenie gazu; FAr (Argon) traktuje się jako stałą część FN_2_ podczas płukania (FAr =FN_2_ x 0,0093 / 0,7881)

Test MBNW z zastosowaniem azotu jako wypłukiwanego gazu obojętnego polega na stopniowym zastępowaniu N_2_ zawartym w powietrzu atmosferycznym, przez 100% O_2_ medyczny. W przypadku testów z zastosowaniem gazów znacznikowych np. SF_6_ należy pamiętać o konieczności przeprowadzenia fazy inhalacji tym gazem (ryc. 4).

Po ustabilizowaniu oddechów pacjenta, następuje zatrzymanie podawania powietrza a tym samym podaży N_2_, natomiast uruchomione zostaje podawanie medycznego 100% O_2_. Stopniowe wypłukiwanie azotu z układu oddechowego, jest prowadzone do momentu osiągnięcia w powietrzu wydychanym 1/40 wartości początkowej stężenia N_2_. Poszczególne etapy badania zostały przedstawione w tabeli I.

**Tabela I:** Etapy testu wypłukiwania azotu metodą wielokrotnych oddechów (opracowanie własne). Table I. Steps of multiple breath nitrogen washout test.

Przygotowanie badania *Preparation of the test*	Przed rozpoczęciem badania należy obligatoryjnie przeprowadzić kalibrację przepływów oraz kalibrację stężenia gazów *At the begining of the test, flow calibration and gas calibration are mandatory*
*Pozycja pacjenta Patient position*	Pacjent wykonuje badanie w pozycji siedzącej, stabilnej z plecami wyprostowanymi, z nogami w miarę możliwości opartymi o podłoże. *The patient should be in a seated position with back straight, with feet resting on the ground*
Sposób oddychania *Breathing*	Pacjent w zależności od możliwości oddycha przez ustnik z klipsem na nosie lub maskę. Oddechy powinny być spokojne, równe i mieścić się w granicach najlepszych wartości pracy czujników. Pacjent nie powinien wykonywać złożonych manewrów oddechowych (śmiech, głębokie wdechy, rozmowa). Bardzo ważne jest zapewnienie przyjaznej atmosfery pozwalającej na prawidłowe wykonanie badania. *The patient breathes through the mouthpiece with a nose clip or through the mask. Breaths should be calm and within the limits of the best value of the sensor*. *The patient should not perform complex breathing maneuvers (laughter, deep breaths, conversation). It is very important to provide a friendly atmosphere that allows you to perform the test properly*.
Animacje *Animations*	W utrzymaniu prawidłowego sposobu oddychania pomagają animacje zaproponowane przez producenta. W przypadku młodszych dzieci zalecane jest oglądanie spokojnych bajek. *Animations help maintain proper breathing. For younger children, it is recommended to watch quiet fairytales*.

Badanie można uznać za wykonane poprawnie w momencie, gdy w co najmniej dwóch prawidłowo przeprowadzonych próbach uzyskano współczynniki LCI 2,5% nieróżniące się więcej niż 5% [10]. Korelacja wyników MBW (Sacin, Scond i LCI – patrz poniżej) może wskazywać na którym poziomie dróg oddechowych doszło do zmian strukturalnych powodujących zwiększoną niejednorodność dystrybucji wentylacji [13].

**Ryc. 3:**
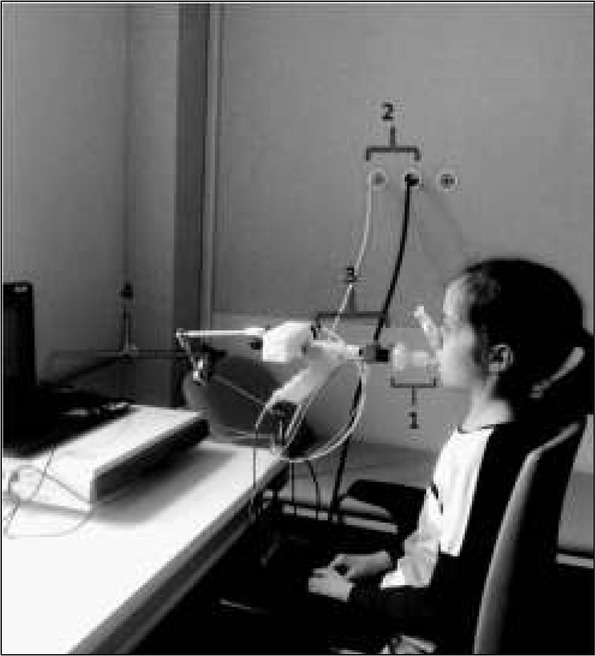
Pacjent podczas testu wypłukiwania azotu metodą wielokrotnych oddechów (materiał własny − zdjęcie wykorzystano za zgodą rodzica). 1 – jednorazowy filtr z ustnikiem, 2 – gazy medyczne powietrze oraz 100% tlen medyczny, 3 – capnostat (analizator CO_2_) wraz z przepływomierzem, 4 – część zabudowana urządzenia z wewnętrznym analizatorem laserowym stężenia O_2_). Fig. 3. Patient during multiple breath nitrogen washout test (own material – photo used with permission from parent). 1 – filter with mouthpiece, 2 – medical gases: air and 100% medical oxygen, 3 – capnostat (CO_2_ analyzer) with flow meter, 4 – part of apparaturs with O_2_ sensor).

**Ryc. 4:**
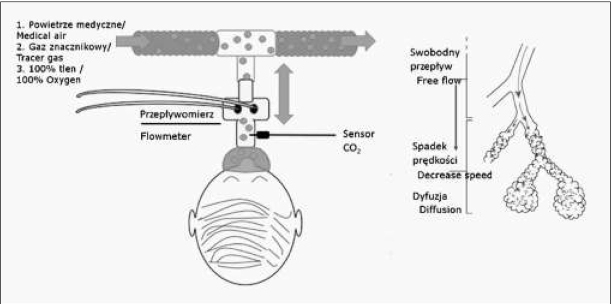
Schemat testu MBW wg [6]. Fig. 4. Schematic illustration of an MBW test [6].

Wyniki pochodzące z badania MBW:

**FRC** – ang. Functional Residual Capacity − czynnościowa pojemność zalegająca. Parametr ten określa ilość powietrza, która pozostaje w płucach po wykonaniu spokojnego wydechu.

**LCI** – ang. Lung Clearance Index – wskaźnik oczyszczania płuc − jest najczęściej stosowanym wskaźnikiem w teście MBW. Współczynnik LCI wyliczany jest ze wzoru:

LCI= CEV/FRC, gdzie

CEV – Cumulative Expired Volume- skumulowana objętość wydechowa, będąca sumą wydychanych objętości oddechowych.

FRC – czynnościowa pojemność zalegająca.

LCI dostarcza informacji o tym, ile razy objętość gazu w płucach na starcie wypłukiwania musi być wymieniona (ang. TO – turnover), aby doprowadzić do wyeliminowania azotu z płuc do 1/40 (2,5%) stężenia wyjściowego. Dotychczasowe badania wykazały, że LCI jest przydatnym wskaźnikiem czynności płuc u chorych na CF, a także ma większą czułość w różnych przedziałach wiekowych w porównaniu z spirometrią [14, 15, 16].

W pełnej analizie wyników badania MBW należy uwzględnić również:

**Scond** (phase III slope index of conductive ventilation inhomogeneity) – wskaźnik zaburzeń w strefie przewodzącej proksymalnej do oskrzelików końcowych.

**Sacin** (phase III slope index of acinar ventilation inhomogeneity ) – wskaźnik zaburzeń wentylacji w pęcherzykach płucnych

**SnIII** (normalized phase III slope) – wskaźniki dysfunkcji wentylacji w najbardziej obwodowych częściach płuc.

Niezwykle istotna jest również ocena kształtu oraz długość krzywej wypłukiwania azotu, a także kształtu krzywej fazy pęcherzykowej (SnIII) [10].

Przykładowe wyniki badania MBNW u pacjentów własnych przedstawiono na rycinie 5.

## Dyskusja

Z naszego doświadczenia wynika, że test wypłukiwania azotu metodą wielokrotnych oddechów (MBNW) jest przyjazną dla pacjenta, nieinwazyjną metodą oceniającą jednorodność dystrybucji wentylacji w drobnych drogach oddechowych poprzez rejestrowanie wypłukiwania obojętnego gazu znacznikowego podczas spokojnego oddychania. W Pracowni Badań Czynnościowych Płuc Centrum Leczenia Mukowiscydozy w Dziekanowie Leśnym badanie to jest wykonywane rutynowo w celu obserwacji zmian współczynnika oczyszczania płuc u pacjentów w wieku od 2 do 28 lat. Należy jednak pamiętać, że LCI jest szeroko stosowanym parametrem oceniającym całkowitą niejednorodność wentylacji w różnych grupach wiekowych od niemowląt do osób starszych. W jednostkach chorobowych, w których dochodzi do zajęcia drobnych dróg oddechowych okazał się być bardziej czułym parametrem niż konwencjonalne badania czynnościowe i lepiej korelującym z uszkodzeniami strukturalnymi płuc. Nowe podejście do analizy krzywych wypłukiwania np. znormalizowana analiza nachylenia fazy III, dostarcza dalszych danych na temat lokalizacji zmian w obrębie drobnych dróg oddechowych i postępu choroby. MBW może być stosowana w diagnostyce i monitorowaniu leczenia wielu chorób, jak mukowiscydoza, astma, zarostowe zapalenie oskrzelików [2, 3, 4, 5]. Poniżej zostaną przedstawione niektóre z nich. Ze względu na ilość dostępnych danych oraz zainteresowania autorów szczególnie szeroko zostanie przedstawione zastosowanie kliniczne MBW w mukowiscydozie.

**Ryc. 5:**
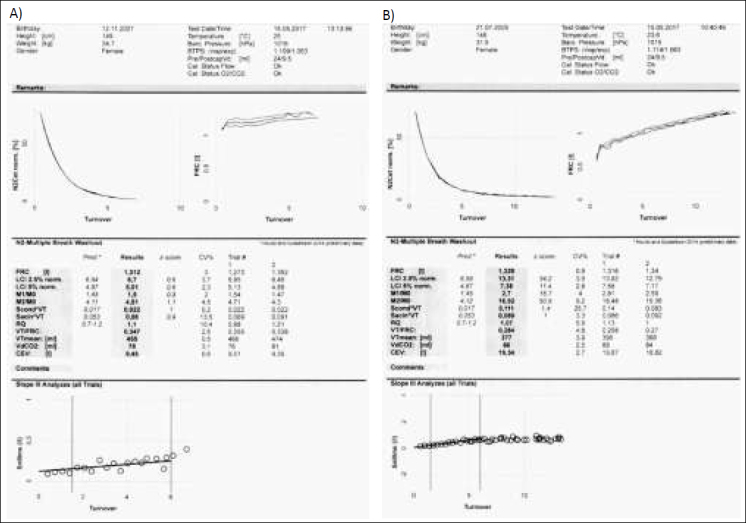
Wyniki badania MBNW chorych na mukowiscydozę: A) wynik w normie B) podwyższony wskaźnik LCI. Fig. 5. Test results MBNW in CF patients: A) result in the standard B) increased LCI.

## Mukowiscydoza

Na przełomie ostatnich dekad oczekiwana długość i jakość życia chorych na mukowiscydozę znacząco wzrosła, nadal jednak powikłania oskrzelowo-płucne pozostają główną przyczyną śmierci. Wraz z coraz lepszą opieką i leczeniem postęp choroby uległ znacznemu spowolnieniu. Należy podkreślić, że choroba płuc rozpoczyna się już w pierwszych miesiącach życia, często jeszcze przed wystąpieniem objawów klinicznych i pogorszeniem wyników spirometrycznych. Jednak ze względu na brak współpracy z pacjentem i metod diagnostycznych dostosowanych do młodego wieku, dopiero od 4-5 rż. rozpoczyna się wykonywanie rutynowych badań czynnościowych płuc oceniających stopień zaawansowania i progresję choroby oskrzelowo-płucnej. Obecnie wiadomo, że FEV 1 nie jest dobrym wskaźnikiem w ocenie wczesnej choroby płuc w CF [2, 3, 4, 17]. Zmiany strukturalne w płucach mogą być obecne u dzieci z prawidłowym wynikiem spirometrii. Pomimo coraz większej liczby dowodów na jego niską czułość, FEV1 pozostaje najczęściej stosowanym parametrem funkcji płuc zarówno w rutynowej ocenie klinicznej, jak i badaniach klinicznych.

Metodą referencyjną do rozpoznawania wczesnych i zaawansowanych zmian strukturalnych w płucach jest tomografia komputerowa o wysokiej rozdzielczości (HRCT ang. high resolution computer tomography). Jednak narażenie na promieniowanie jonizujące ogranicza jego wykorzystanie jako narzędzia do monitorowania przebiegu choroby. W ostatnich latach wzrosło zainteresowanie HRCT jako metody oceny wczesnego stadium choroby oskrzelowo-płucnej, która nie jest wykrywalna w konwencjonalnej spirometrii. Również próby stosowania HRCT jako punktu końcowego w badaniach klinicznych u małych dzieci z CF wydają się obiecujące. W celu zminimalizowania dawki kumulacyjnej opracowano protokoły o zmniejszonej dawce promieniowania jonizującego.

Wykonywanie HRCT u młodszych pacjentów jest jednak czasochłonne i obciążające. To procedura wymagająca sedacji lub znieczulenia ogólnego, powodująca wzrost niepokoju i lęku u pacjentów i ich rodzin. Natomiast MBW jest nieinwazyjną, bezpieczną i czułą metodą oceniającą wczesny okres choroby płuc w CF [[2, 3, 4, 14, 15, 18]. Kilka wskaźników takich jak LCI można wyliczyć na podstawie krzywych wypłukiwania opisujących obecność i zakres niehomogenności wentylacji. LCI odzwierciedla VI w zakresie obwodowych i przewodzących dróg oddechowych. W porównaniu do spirometrii znaczna część pacjentów ma nieprawidłowy wynik LCI, nawet jeśli FEV1 >80% w.nal. [6, 19, 20, 21]

Ostatnio stwierdzono, że parametry niehomogeniczności wentylacji mierzone podczas MBW mogą być porównywalne do HRCT w diagnostyce zmian płucnych u pacjentów z CF [18]. LCI ma wysoką czułość w wykrywaniu nieprawidłowości strukturalnych. Szczególnie pacjenci z wczesną chorobą płuc mogą odnieść korzyść z wprowadzenia MBW jako czułego, nieinwazyjnego narzędzia klinicznego. Udowodniono wartość diagnostyczną LCI jako wskaźnika porównywalnego do niskodawkowej HRCT w wykrywaniu wczesnych zmian płucnych u dzieci, nastolatków i młodych dorosłych z CF i prawidłowym FEV1 (>80%w.nal.).

Metoda ta pozwala na wczesne rozpoznanie, monitorowanie choroby płuc oraz ocenę wyników stosowania nowych strategii leczenia w celu zatrzymania lub spowolnienia progresji zmian płucnych. MBW jest równie czułą metodą co niskodawkowa HRCT w wykrywaniu wczesnych zmian płucnych u młodych pacjentów z CF i może być stosowana jako nieinwazyjna metoda zarówno w rutynowej opiece, jak i w badaniach klinicznych.

Badanie MBW weszło już w schemat corocznych badań bilansowych w niektórych ośrodkach mukowiscydozy. Zgodnie z zaleceniami LCI powinno stanowić rutynową część corocznej oceny i być wykonywane u wszystkich dzieci w wieku ≥5 lat [22].

Możliwość rozpoznawania wczesnych zaburzeń dróg oddechowych w tych „cichych latach,” kiedy FEV1 mieści się w granicach normy, jest szczególnie przydatne w badaniu nowych terapii u niemowląt i małych dzieci z łagodną chorobą oskrzelowo-płucną [2]. LCI zaczyna być stosowany jako punkt końcowy w badaniach klinicznych w mukowiscydozie m.in z najnowszymi lekami jak ivacaftor lub też w badaniach interwencyjnych np. z użyciem rhDNazy oraz soli hipertonicznej [23].

W ośrodku autorów od stycznia 2017 r. prowadzone są badania oceniające przydatność testu MBNW w monitorowaniu przebiegu choroby oskrzelowo-płucnej u pacjentów z mukowiscydozą. Pod opieką Centrum Leczenia Mukowiscydozy w Dziekanowie Leśnym pozostaje 400 pacjentów od wieku niemowlęcego do 18 rż. Chorzy mają wykonywane badania czynnościowe układu oddechowego podczas badań bilansowych oraz przed i po leczeniu zaostrzeń zmian oskrzelowo-płucnych. W fazie badań wstępnych pozostaje korelacja zaburzeń homogenności wentylacji (LCI) z innymi badaniami jak spirometria, bodypletyzmografia, oscylometria oraz wpływ leczenia zaostrzeń zmian oskrzelowo-płucnych na jego zmienność. Niemniej interesującym zagadnieniem jest zależność przewlekłego zakażenia dróg oddechowych florą patogenną w tym przede wszystkim pałeczką ropy błękitnej (Pseudomonas aeruginosa) oraz gronkowcem złocistym (Staphylococcus aureus) i nasilenia zaburzeń wentylacji płuc u chorych na mukowiscydozę. Po podsumowaniu wyników, badania te będą one przedmiotem kolejnej pracy.

## Astma oskrzelowa

Astma oskrzelowa jest przewlekłą chorobą zapalną całego drzewa oskrzelowego, nie tylko dużych oskrzeli ale i drobnych dróg oddechowych. Potwierdzają to badania histopatologiczne, w których stwierdza się m.in. zwiększoną liczbę komórek zapalnych w obrębie drobnych dróg oddechowych i przestrzeni pęcherzykowych. Dane te wskazują na duży potencjał nieinwazyjnych badań oceniających czynność drobnych dróg oddechowych, nie tylko ułatwiających rozpoznanie astmy szczególnie u młodszych dzieci, lecz także ocenę i monitorowanie jej przebiegu [3, 4, 5].

Udział drobnych dróg oddechowych w astmie został potwierdzony w wielu badaniach. Całkowity wzrost niehomogenności wentylacji (VI) wyrażony wzrostem LCI stwierdzono u chorych na astmę w porównaniu z grupą kontrolną. Badania IGW ( zarówno MBW jak i SBW) potwierdziły przewlekłe zmiany patologiczne części przewodzącej układu oddechowego [24]. U dzieci i nastolatków z astmą, u których stwierdzono prawidłowy wynik spirometrii przy jednoczesnej obecności patologicznej pułapki powietrznej, potwierdzono udział drobnych dróg oddechowych w patogenezie zmian. U chorych na astmę obserwowano zwiększenie pułapki powietrznej w pozycji leżącej w porównaniu do siedzącej, co może tłumaczyć mechanizm występowania objawów nocnych [1].

Niehomogenność wentylacji (VI) wydaje się być ważnym czynnikiem predykcyjnym nadreaktywności dróg oddechowych w astmie niezależnie od stanu zapalnego [25]. Jest ona zgodna z badaniami obrazowymi wykrywającymi niejednolite zaburzenia wentylacji zarówno przed jak i po testach prowokacji. Charakter mechanizmów zależności pomiędzy VI, nadreaktywniością dróg oddechowych i zapaleniem pozostaje nadal niejasny [26].

IGW może być bardziej czułym narzędziem diagnostycznym w rozpoznawaniu astmy niż spirometria. LCI i Scond różnicowały nawracające świsty wieku przedszkolnego od zdrowej grupy kontrolnej [27] w przeciwieństwie do swoistego oporu dróg oddechowych (sRaw ang. specific airway resistance). Ponadto w testach prowokacyjnych można wykryć odpowiedź obwodowych dróg oddechowych [28]. Dodatkową wartością IGW w monitorowaniu astmy jest nachylenie SBW fazy III różnicujące pacjentów z częstymi zaostrzeniami od tych ze stabilną astmą [29].

MBW może być również użytecznym nieinwazyjnym markerem przebudowy dróg oddechowych (remodelingu). W grupie dorosłych z łagodną astmą udokumentowano wzrost Scond, nie w pełni odwracalny po zastosowaniu leku rozszerzającego oskrzela. W grupie chorych na astmę łagodną do umiarkowanej udowodniono wzrost zaburzeń wentylacji w gronkach z nieodwracalnym wzrostem Sacin. Dane te są zgodne z ewolucją procesów naprawczych drobnych dróg oddechowych i mogą wskazywać na remodeling dróg oddechowych. Badania histologiczne dużych dróg oddechowych sugerują, że proces ten rozpoczyna się w młodym wieku i został udokumentowany u dzieci w wieku szkolnym. Wzrost LCI był tylko częściowo odwracalny w stabilnej, łagodnej i przetrwałej astmie dziecięcej [30] i może stanowić pierwszy objaw przebudowy dróg oddechowych.

Poprawę w Sacin bez zmian w Scond wykazano u dorosłych chorych na astmę po zmianie preparatu glikokortykosteroidu wziewnego na zawierający bardzo małe cząsteczki [31]. Pacjenci z nieprawidłowym Sacin mieli niższe wartości FEV1 niż ci z prawidłowymi. Sugeruje się, że leczenie lekami zawierającymi bardzo małe cząsteczki może być korzystne u chorych na astmę z zajęciem bardziej obwodowych dróg oddechowych.

Jak przedstawiono, zastosowanie MBW u chorych na astmę może poprawić zrozumienie procesów patofizjologicznych, szczególnie dotyczących nadreaktywności oskrzeli i dostarczyć dodatkowego narzędzia do oceny odpowiedzi klinicznej na stosowane leczenie. Wysoka czułość badania stwarza możliwość wczesnego rozpoznania i leczenia, a w przyszłości poprawy rokowania [3, 4, 5].

## Dysplazja oskrzelowo-Płucna

Postęp w leczeniu BPD (ang. bronchopulmonary dysplasia), wprowadzenie surfaktantu oraz różnych metod wentylacji, spowodował wyodrębnienie dwóch populacji chorych na BPD: starą” − przed stosowaniem surfaktantu i „nową” − po jego wprowadzeniu surfaktantu. Obecnie dzieci urodzone przedwcześnie z przewlekłą chorobą płuc prezentują odmienny obraz kliniczny i patomorfologiczny niż te sprzed „ery surfaktantu”. Mimo, że w obu typach dochodzi do przerwania wewnątrzłonowego rozwoju płuc, to „stara” BPD charakteryzuje się m.in. intensywnym, rozlanym włóknieniem przegród pęcherzykowych, utrzymującym się aż do wczesnego wieku przedszkolnego. Nowe „BPD” charakteryzuje się łagodniejszym, bardziej rozproszonym procesem włóknienia. Sugeruje to, że w przebiegu BPD dochodzi do mieszanych zaburzeń restrykcyjno-obturacyjnych. W zależności od sposobu postępowania w okresie noworodkowym różny jest stopień obturacji dróg oddechowych, a tym samym zaburzeń homogenności wentylacji [4].

Wstępne badania niemowląt z BPD opisują wzrost niejednorodności wentylacji i obniżenie FRC [32]. Stwierdza się korelację tych zmian z ciężkością choroby. W innym badaniu przedstawiono, że samo wcześniactwo spowodowało obniżenie FRC i wzrost niehomogenności wentylacji (VI) [33]. Wyniki badań są sprzeczne. Konieczne jest przeprowadzenie dalszych badań celem oceny przydatności MBW u dzieci z BPD.

Przydatność MBW w BPD pozostaje niejasna. Biorąc pod uwagę przeważające zaburzenia restrykcyjne oraz rozproszony charakter zmian w „nowym” BPD, przydatność MBW może być niewielka w stosunku do tradycyjnych badań [3, 4, 5].

## Zarostowe zapalenie oskrzelików

Zarostowe zapalenie oskrzelików (BO − ang. bronchiolitis obliterans) jest chorobą zapalną drobnych dróg oddechowych, przebiegającą w sposób niejednorodny z zwłóknieniem i zanikiem dystalnych dróg oddechowych. Może być spowodowana infekcją wirusową, uszkodzeniem chemicznym lub zaburzeniami odporności. Badania sugerują przydatność MBW we wczesnym wykrywaniu BO zarówno po transplantacji płuc [34] jak i po przeszczepieniu komórek macierzystych [35]. Dane pediatryczne są obecnie ograniczone do kilku opisów przypadków [36]. Rozpoznanie i intensywne leczenie we wczesnym stadium zapalenia może zapobiec postępowi oraz znacznej zachorowalności i śmiertelności związanej z tym rozpoznaniem.

## Pierwotna dyskineza rzęsek (PCD − ang. Primary ciliary dyskinesia)

Pierwotna dyskineza rzęsek i mukowiscydoza to choroby o autosomalnie recesywnym typie dziedziczenia, z dominującymi objawami klinicznymi ze strony układu oddechowego. Obie choroby charakteryzują się zaburzeniem klirensu śluzowo-rzęskowego, przewlekłym bakteryjnym zakażeniem dróg oddechowych, zapaleniem neutrofilowym i stopniową progresją zmian oskrzelowo-płucnych. Etiologia tych zaburzeń jest jednak odmienna. Do zaburzeń klirensu śluzowo-rzęskowego w PCD dochodzi w wyniku nieprawidłowej budowy lub /i funkcji aparatu rzęskowego. Przebieg choroby jest znacznie łagodniejszy niż CF a czas przeżycia znacznie dłuższy.

Analogicznie do wyników badań u chorych na mukowiscydozę, u których stwierdza się korelację wskaźnika LCI z nieprawidłowościami w HRCT klatki piersiowej, zakładano, że badanie MBW będzie również przydatne do oceny choroby płuc w pierwotnej dyskinezie rzęsek. Zbadano zależność pomiędzy LCI, wynikami spirometrii i HRCT u chorych w zaawansowanym stadium PCD. W przeciwieństwie do pacjentów z CF w badanej grupie chorych nie stwierdzono korelacji pomiędzy FEV1 a LCI ani pomiędzy HRCT, LCI i FEV1. Wysunięto przypuszczenie, ze różnica w korelacji wynika być może z odmienności zajęcia dużych i drobnych dróg oddechowych w tych dwóch jednostkach chorobowych [37].

Z kolei w innym badaniu prospektywnym porównywano LCI z FEV1 z wynikami badań obrazowych- HRCT klatki piersiowej u chorych z łagodną do umiarkowanej PCD. Wartość LCI korelowała z wynikiem HRCT i ze zmianami takimi jak pogrubienie ścian oskrzeli, korki śluzowe i rozstrzenie oskrzeli. W porównaniu z FEV 1 wskaźnk LCI był bardziej czułym parametrem w wykrywaniu nieprawidłowości strukturalnych. Udowodniono, że pomiar LCI u chorych na PCD ma znaczenie kliniczne i jest czulszym wskaźnikiem korelującym z nieprawidłowościami strukturalnymi w HRCT niż FEV1[38].

## Wrodzona przepuklina przeponowa z hipoplazją płuc

Wrodzona przepuklina przeponowa (CDH − ang. congenital diaphragmatic hernia) powodując zmniejszenie się przestrzeni wewnątrz klatki piersiowej w okresie alweolaryzacji, prowadzi do rozwoju hipoplazji płuc. Badania przeprowadzone u dzieci w wieku szkolnym wskazywały na zaburzenia o typie obturacji o różnym stopniu ciężkości [39]. W badaniu niemowląt z CDH stwierdzono wzrost niehomogenności wentylacji [40]. Przydatność MBW w CDH podobnie jak w BPD może być związana z oceną komponenty zaburzeń o typie obturacji w przebiegu choroby.

## Neuroendokrynna hiperplazja u niemowląt (NEHI − ang. Neuroendocrine cell hyperplasia of infancy)

Niemowlęta z przetrwałym tachypnoe mogą prezentować różne zaburzenia oddechowo-krążeniowe. MBW może ułatwić różnicowanie zaburzeń obturacjnych drobnych dróg oddechowych jak grudkowe zapalenie oskrzelików (ang Follicular bronchitis − FB) od chorób restrykcyjnych z prawidłowym LCI i obniżonym FRC [5]. Potrzebne są dalsze badania w tej grupie pacjentów celem oceny przydatności MBW w diagnostyce i monitorowaniu choroby.

## Wnioski i przyszłe kierunki

Rozwój zmian patologicznych w przebiegu wielu chorób drobnych dróg oddechowych obserwowany jest już u najmłodszych dzieci. W celu ograniczenia lub zapobiegania uszkodzeniu płuc ogromne znaczenie mają wczesne rozpoznanie i rozpoczęcie właściwego leczenia. Wzrost zainteresowania MBW jako badania zapewniającego wgląd w procesy patologiczne drobnych dróg oddechowych przyczynił się do coraz częstszego jego stosowania. Zostało ono uznane za czułe, bezpieczne i przydatne narzędzie do badania funkcji płuc i ich odpowiedzi na różne czynniki uszkadzające. Z naszego doświadczenia wynika, że poprzez ocenę niejednorodności wentylacji płuc daje możliwość rozpoznania zaburzeń już w początkowym stadium wielu chorób, często kiedy jeszcze wyniki konwencjonalnych badań spirometrycznych są prawidłowe. To proste badanie wymagające od pacjenta spokojnego oddychania, bez konieczności wykonywania forsownych oddechów, pozwala na bezinwazyjną ocenę drobnych dróg oddechowych w różnych grupach wiekowych : od populacji dziecięcej po osoby dorosłe źle współpracujące. Obecnie dąży się do standaryzacji wyników i określenia norm przydatnych w diagnostyce, monitorowaniu przebiegu i leczenia chorób drobnych dróg oddechowych. Zastosowanie testów oceniających funkcję płuc u niemowląt i dzieci przedszkolnych z mukowiscydozą i innymi chorobami drobnych dróg oddechowych może zmodyfikować postępowanie kliniczne i poprawić rokowanie [3, 4, 5]. W ośrodku autorów prowadzone są dalsze badania dotyczące wykorzystania tej metody u chorych na mukowiscydozę będące tematem kolejnej pracy.
